# Obstetric Management of COVID-19 in Pregnant Women

**DOI:** 10.3389/fmicb.2020.01186

**Published:** 2020-05-26

**Authors:** Youwen Mei, Dan Luo, Sumei Wei, Xiaoyan Liao, Yue Pan, Xiao Yang, Yonghong Lin

**Affiliations:** Department of Obstetrics and Gynecology, Chengdu Women and Children's Central Hospital Affiliated to University of Electronic Science and Technology of China, Chengdu, China

**Keywords:** COVID-19, perinatal outcome, antiviral drug, vertical transmission, severe acute respiratory syndrome, Middle East respiratory syndrome, obstetric management

## Abstract

The 2019 novel coronavirus disease (COVID-19), which is caused by the novel beta coronavirus, SARS-CoV-2, is currently prevalent all over the world, causing thousands of deaths with relatively high virulence. Like two other notable beta coronaviruses, severe acute respiratory syndrome coronavirus-1 (SARS-CoV-1) and Middle East respiratory syndrome coronavirus (MERS-CoV), SARS-CoV-2 can lead to severe contagious respiratory disease. Due to impaired cellular immunity and physiological changes, pregnant women are susceptible to respiratory disease and are more likely to develop severe pneumonia. Given the prevalence of COVID-19, it is speculated that some pregnant women have already been infected. However, limited data are available for the clinical course and management of COVID-19 in pregnancy. Therefore, we conducted this review to identify strategies for the obstetric management of COVID-19. We compared the clinical course and outcomes of COVID-19, SARS, and MERS in pregnancy and discussed several drugs for the treatment of COVID-19 in pregnancy.

## Introduction

The COVID-19 outbreak emerged in 2019. The causative pathogen for COVID-19 was identified as a novel beta coronavirus, SARS-CoV-2, which is genetically related to severe acute respiratory syndrome coronavirus-1 (SARS-CoV-1) and Middle East respiratory syndrome coronavirus (MERS-CoV; Lu et al., 2020). Furthermore, SARS-CoV-2 and SARS-CoV-1 share the same cellular receptor, angiotensin-converting enzyme 2 (ACE2), despite amino acid variation at some key residues (Chan et al., 2020). All three coronaviruses can cause severe infectious respiratory disease. Currently, COVID-19 has spread across multiple countries (Holshue et al., 2020; Hui et al., 2020), and it is reasonable to speculate that some pregnant women have been infected. However, little is known about the clinical course and the obstetric management of COVID-19 in pregnancy. Available data could be drawn from cases of pregnancy complicated with SARS and MERS. This review was conducted to investigate the clinical course and outcome of COVID-19 in pregnancy and to discuss several drugs that could be used for pregnant women with COVID-19.

## Comparison of COVID-19, SARS, and MERS In pregnancy

We searched PubMed and the China National Knowledge Infrastructure Database for literature that was published up to March 30, 2020, using the keywords “coronavirus,” “pregnant,” or “neonate.” Data about pregnancy with COVID-19, SARS, and MERS were extracted and compared by gestational age. Table 1 shows maternal clinical characteristics, and Table 2 shows treatment and perinatal outcomes of the three coronavirus infections in pregnancy.

**Table 1:** Maternal clinical and laboratory characteristics of COVID-19, SARS, and MERS in pregnancy.

**References**	**Patient no. (disease + number)**	**Trimester**	**Nationality**	**Age (years)**	**GA on Admission (weeks)**	**Fever**	**Cough**	**Myalgia**	**Malaise**	**Dyspnea**	**Signs of viral pneumonia on CT**	**Lymphopenia**
Zhao et al. (2020)	COVID-19-1	2nd	China	38	25 + 5	Yes	Yes	No	Yes	Yes	Yes	Yes
Chen H. et al. (2020)	COVID-19-2	3rd	China	33	37 + 2	No	Yes	No	No	No	Yes	No
Chen H. et al. (2020)	COVID-19-3	3rd	China	27	38 + 2	Yes	Yes	Yes	No	No	Yes	Yes
Chen H. et al. (2020)	COVID-19-4	3rd	China	40	36	No	Yes	No	No	No	Yes	Yes
Chen H. et al. (2020)	COVID-19-5	3rd	China	26	36 + 2	Yes	No	No	No	Yes	Yes	No
Chen H. et al. (2020)	COVID-19-6	3rd	China	26	38 + 1	Yes	No	Yes	Yes	No	Yes	Yes
Chen H. et al. (2020)	COVID-19-7	3rd	China	26	36 + 3	Yes	Yes	Yes	Yes	No	Yes	Yes
Chen H. et al. (2020)	COVID-19-8	3rd	China	29	36 + 2	Yes	No	No	No	No	No	Yes
Chen H. et al. (2020)	COVID-19-9	3rd	China	28	38	Yes	No	No	No	No	Yes	No
Chen H. et al. (2020)	COVID-19-10	3rd	China	34	39 + 4	Yes	No	No	No	No	Yes	No
Yu et al. (2020)	COVID-19-11	3rd	China	34	39 + 6	Yes	No	No	No	No	Yes	Yes
Yu et al. (2020)	COVID-19-12	3rd	China	30	38 + 5	Yes	No	No	No	Yes	Yes	No
Yu et al. (2020)	COVID-19-13	3rd	China	31	41 + 2	Yes	No	No	No	No	Yes	Yes
Yu et al. (2020)	COVID-19-14	3rd	China	33	37	No	Yes	No	No	No	Yes	Yes
Yu et al. (2020)	COVID-19-15	3rd	China	29	40 + 4	Yes	No	No	No	No	Yes	No
Yu et al. (2020)	COVID-19-16	3rd	China	34	38 + 2	Yes	No	No	No	No	Yes	Yes
Yu et al. (2020)	COVID-19-17	3rd	China	34	38 + 4	Yes	No	No	No	No	Yes	Yes
Zhu et al. (2020)	COVID-19-18	3rd	China	25	—	Yes	No	No	No	No	Yes	—
Zhu et al. (2020)	COVID-19-19	3rd	China	35	—	Yes	No	No	No	No	Yes	—
Zhu et al. (2020)	COVID-19-20	3rd	China	35	—	No	Yes	No	No	No	Yes	—
Zhu et al. (2020)	COVID-19-21	3rd	China	30	—	Yes	No	No	No	No	Yes	—
Zhu et al. (2020)	COVID-19-22	3rd	China	30	—	Yes	No	No	No	No	Yes	—
Zhu et al. (2020)	COVID-19-23	3rd	China	30	—	Yes	Yes	No	No	No	Yes	—
Zhu et al. (2020)	COVID-19-24	3rd	China	30	—	Yes	Yes	No	No	No	Yes	—
Zhu et al. (2020)	COVID-19-25	3rd	China	29	—	Yes	Yes	No	No	No	Yes	—
Zhu et al. (2020)	COVID-19-26	3rd	China	34	—	Yes	No	No	No	No	Yes	—
Chen S. et al. (2020)	COVID-19-27	3rd	China	23	37 + 3	Yes	No	No	No	Yes	Yes	No
Chen S. et al. (2020)	COVID-19-28	3rd	China	34	38 + 6	Yes	No	No	No	No	Yes	No
Chen S. et al. (2020)	COVID-19-29	3rd	China	32	35	Yes	No	No	No	No	Yes	No
Wong et al. (2004)	SARS-1	1st	China	33	4	Yes	Yes	Yes	Yes	No	Yes	No
Wong et al. (2004)	SARS-2	1st	China	34	4	Yes	No	Yes	Yes	No	Yes	No
Wong et al. (2004)	SARS-3	1st	China	44	4	Yes	Yes	Yes	Yes	Yes	Yes	Yes
Wong et al. (2004)	SARS-4	1st	China	24	3	Yes	Yes	Yes	No	No	Yes	No
Wong et al. (2004)	SARS-5	1st	China	24	6	Yes	No	Yes	Yes	No	Yes	Yes
Wong et al. (2004)	SARS-6	1st	China	25	12	Yes	Yes	Yes	Yes	Yes	Yes	Yes
Wong et al. (2004)	SARS-7	1st	China	26	3	Yes	Yes	Yes	Yes	No	Yes	Yes
Wong et al. (2004)	SARS-8	2nd	China	32	26	Yes	No	Yes	Yes	No	Yes	No
Zhang et al. (2003)	SARS-9	2nd	China	34	27	Yes	Yes	Yes	Yes	Yes	Yes	Yes
Zhang et al. (2003)	SARS-10	2nd	China	—	25 + 2	Yes	Yes	No	Yes	No	Yes	Yes
Robertson et al. (2004)	SARS-11	2nd	—	—	15 + 2	Yes	Yes	No	No	No	Yes	Yes
Yudin et al. (2005)	SARS-12	2nd	Canada	36	19	Yes	Yes	No	No	Yes	Yes	Yes
Wong et al. (2004)	SARS-13	3rd	China	33	31	Yes	Yes	No	No	No	Yes	Yes
Wong et al. (2004)	SARS-14	3rd	China	27	28	Yes	Yes	Yes	Yes	Yes	Yes	Yes
Wong et al. (2004)	SARS-15	3rd	China	30	30	Yes	Yes	Yes	Yes	No	Yes	Yes
Wong et al. (2004)	SARS-16	3rd	China	34	32	Yes	Yes	Yes	Yes	No	Yes	Yes
Zhang et al. (2003)	SARS-17	3rd	China	—	30 + 2	Yes	Yes	Yes	Yes	Yes	Yes	Yes
Zhang et al. (2003)	SARS-18	3rd	China	—	36	Yes	Yes	No	No	No	Yes	Yes
Zhang et al. (2003)	SARS-19	3rd	China	—	40 + 4	Yes	No	No	No	No	Yes	Yes
Alfaraj et al. (2019)	MERS-1	1st	Saudi Arabia	29	6	No	No	No	No	No	—	—
Payne et al. (2014)	MERS-2	2nd	Jordanian	39	5 months	Yes	Yes	No	Yes	No	—	—
Assiri et al. (2016)	MERS-3	2nd	Saudi Arabia	27	22	Yes	Yes	No	No	Yes	Yes	—
Assiri et al. (2016)	MERS-4	2nd	Saudi Arabia	30	23	Yes	Yes	No	No	Yes	—	—
Alfaraj et al. (2019)	MERS-5	2nd	Saudi Arabia	39	24	No	No	No	No	No	—	—
Assiri et al. (2016)	MERS-6	2nd	Saudi Arabia	31	24	No	Yes	Yes	No	Yes	Yes	—
Alserehi et al. (2016)	MERS-7	3rd	Saudi Arabia	33	31	Yes	Yes	No	No	Yes	Yes	—
Malik et al. (2016)	MERS-8	3rd	United Arab Emirates	32	32	Yes	Yes	No	No	Yes	Yes	—
Assiri et al. (2016)	MERS-9	3rd	Saudi Arabia	34	34	No	No	No	No	Yes	Yes	—
Park et al. (2016)	MERS-10	3rd	South Korean	39	35 + 4	No	No	Yes	No	No	Yes	—
Assiri et al. (2016)	MERS-11	3rd	Saudi Arabia	32	38	Yes	Yes	No	No	Yes	Yes	—

*GA, gestational age; COVID-19, 2019 novel coronavirus disease; SARS, severe acute respiratory syndrome coronavirus; MERS, Middle East Respiratory Syndrome coronavirus; CT, computed tomography; —, no data*.

**Table 2:** Treatment and perinatal outcomes of COVID-19, SARS, and MERS in pregnancy.

**Patient no**.	**GA of delivery**	**Antiviral therapy**	**Antibiotic therapy**	**Use of corticosteroid**	**Delivery mode**	**Complication during pregnancy**	**Indication of C-section**	**Maternal outcome**	**Neonatal outcome**
COVID-19-1		Yes	No	Yes	—	—	—	Survived	Ongoing pregnancy
COVID-19-2	37 + 3	Yes	Yes	No	Cesarean section	Severely elevated liver enzymes	COVID-19	Survived	Survived
COVID-19-3	39 + 1	Yes	Yes	No	Cesarean section	—	COVID-19	Survived	Survived
COVID-19-4	36 + 4	Yes	Yes	No	Cesarean section	—	History of C-section (×2); COVID-19	Survived	Survived
COVID-19-5	36 + 5	No	Yes	No	Cesarean section	Pre-eclampsia	Pre-eclampsia; COVID-19	Survived	Survived
COVID-19-6	38 + 2	No	Yes	No	Cesarean section	Fetal distress	Fetal distress; COVID-19	Survived	Survived
COVID-19-7	37	No	Yes	No	Cesarean section	—	History of stillbirth (×2); COVID-19	Survived	Survived
COVID-19-8	36 + 4	Yes	Yes	No	Cesarean section	PROM	COVID-19	Survived	Survived
COVID-19-9	38 + 2	Yes	Yes	No	Cesarean section	Fetal distress	Fetal distress; COVID-19	Survived	Survived
COVID-19-10	40 + 3	Yes	Yes	No	Cesarean section	PROM	PROM; COVID-19	Survived	Survived
COVID-19-11	40	Yes	Yes	Yes	Cesarean section	—	Mature; COVID-19	Survived	Survived
COVID-19-12	38 + 5	Yes	Yes	Yes	Cesarean section	Elevated liver enzymes	Mature; Precious fetus; Placentas racquet	Survived	Survived
COVID-19-13	41 + 5	Yes	Yes	Yes	Cesarean section	Fetal distress	Mature; Fetal distress; COVID-19	Survived	Survived
COVID-19-14	37	Yes	Yes	Yes	Cesarean section	—	Mature; History of C-section	Survived	Survived
COVID-19-15	40 + 6	Yes	Yes	No	Cesarean section	—	Mature; Failure trial vaginal	Survived	Survived
COVID-19-16	38 + 2	Yes	Yes	Yes	Cesarean section	—	Mature; Parturient History of C-section;	Survived	Survived
COVID-19-17	38 + 4	Yes	Yes	No	Cesarean section	Elevated liver enzymes	Mature; History of C-section; COVID-19	Survived	Survived
COVID-19-18	38 + 4	—	—	—	Cesarean section	Fetal distress; Oligohydramnios	—	Survived	Survived
COVID-19-19	33 + 6	—	—	—	Cesarean section	PROM	—	Survived	Survived
COVID-19-20	34 + 2	—	—	—	Vaginal delivery	PROM; fetal distress	—	Survived	Survived
COVID-19-21	34 + 5	—	—	—	Cesarean section	—	—	Survived	died
COVID-19-22	39	—	—	—	Cesarean section	Cholecystitis; fetal distress	—	Survived	Survived
COVID-19-23	37	—	—	—	Cesarean section	Fetal distress; polyhydramnios	—	Survived	Survived
COVID-19-24	34 + 6	—	—	—	Cesarean section	Fetal distress	—	Survived	Survived
COVID-19-25	31 (Twin)	—	—	—	Vaginal delivery	Fetal distress	—	Survived	Survived
COVID-19-26	39	—	—	—	Cesarean section	—	—	Survived	Survived
COVID-19-27	37 + 4	—	—	—	Cesarean section	Complete placenta previa	—	Survived	Survived
COVID-19-28	39	—	—	—	Cesarean section	Acute cholecystitis, placental abruption	—	Survived	Survived
COVID-19-29	35	—	—	—	Cesarean section	Complete placenta previa	—	Survived	Survived
SARS-1	2	Yes	Yes	Yes	—	—	—	Survived	spontaneous abortion
SARS-2	3	Yes	Yes	Yes	—	—	—	Survived	Spontaneous abortion
SARS-3	5	Yes	Yes	Yes	—	—	—	Died	Spontaneous abortion
SARS-4	3	Yes	Yes	Yes	—	—	—	Survived	Spontaneous abortion
SARS-5	1	Yes	Yes	Yes	—	—	—	Survived	Artificial abortion
SARS-6	1	Yes	Yes	Yes	—	—	—	Survived	Artificial abortion
SARS-7	3	No	Yes	No	—	—	—	Survived	Ongoing pregnancy
SARS-8	26	Yes	Yes	Yes	Cesarean section	Severe condition	Severe condition	Survived	Survived
SARS-9	28	Yes	Yes	Yes	Cesarean section	Severe condition	Severe condition	Died	Survived
SARS-10	38 + 4	No	No	No	Cesarean section	Meconium-stained amniotic fluid	Social factor	Survived	Survived
SARS-11	33 + 6	No	No	No	Cesarean section	Fetal distress; elevated liver enzymes	Fetal distress	Survived	Survived
SARS-12	38	Yes	Yes	No	Cesarean section	Complete placenta previa	Complete placenta previa	Survived	Survived
SARS-13	38	No	Yes	Yes	Vaginal delivery	—	—	Survived	Survived
SARS-14	33	Yes	Yes	Yes	Vaginal delivery	DIC; IUGR; oligohydramnios; preterm labor	—	Survived	Survived
SARS-15	37	Yes	Yes	Yes	Cesarean section	Renal failure; IUGR; oligohydramnios	Intrapartum nonreassuring CTG	Survived	Survived
SARS-16	32	Yes	Yes	Yes	Cesarean section	Severe condition	Severe condition	Died	Survived
SARS-17	31 + 5	No	No	No	Cesarean section	Elevated liver enzymes; fetal distress	Fetal distress	Survived	Survived
SARS-18	38 + 2	No	No	No	Cesarean section	Fetal distress; elevated liver enzymes	Fetal distress	Survived	Survived
SARS-19	40 + 5	No	No	No	Cesarean section	Fetal distress; elevated liver enzymes	Fetal distress	Survived	Survived
MERS-1	Term	—	—	—	—	—	—	Survived	Ongoing pregnancy
MERS-2	Stillbirth	—	—	—	—	—	—	Survived	Died
MERS-3	Term	—	—	—	—	—	—	Survived	Survived
MERS-4	Term	—	—	—	—	—	—	Survived	Survived
MERS-5	Term	—	—	—	—	—	—	Survived	Survived
MERS-6	24	—	—	—	—	—	—	Died	Died
MERS-7	32	Yes	Yes	Yes	—	—	—	Survived	Survived
MERS-8	32	Yes	Yes	No	—	—	—	Died	Survived
MERS-9	Stillbirth	—	—	—	—	—	—	Survived	Died
MERS-10	37 + 4	No	No	No	—	—	—	Survived	Survived
MERS-11	Term	—	—	—	—	—	—	Died	Survived

*GA, gestational age; COVID-19, 2019 novel coronavirus disease; SARS, severe acute respiratory syndrome coronavirus; MERS, Middle East Respiratory Syndrome coronavirus; CT, computed tomography; CTG, cardiotocography; PROM, premature rupture of membranes; DIC, disseminated intravascular coagulation; IUGR, intrauterine growth retardation; —, no data*.

Twenty-nine cases of COVID-19 during pregnancy were found in five reports (Chen H. et al., 2020; Chen S. et al., 2020; Yu et al., 2020; Zhao et al., 2020; Zhu et al., 2020). One patient was infected in the second trimester (Zhao et al., 2020), and the others in the third trimester (Chen H. et al., 2020; Chen S. et al., 2020; Yu et al., 2020; Zhu et al., 2020). The pregnant woman infected in the second trimester had symptoms of fever, cough, malaise, and subsequently dyspnea. Blood counts showed lymphopenia, and computed tomography (CT) revealed diffused bilateral infiltrates in the lungs. She was given lopinavir/ritonavir, interferon α-2b, methylprednisolone, abidol, and gamma globulin successively. After 18 days of treatment, her condition improved, and virus nucleic acid testing for SARS-CoV-2 was negative twice. Thus, she continued with the pregnancy. On the other hand, the main symptoms of the other 28 patients infected in the third trimester were fever (89.3%), cough (35.7%), and myalgia (14.3%). CT images of 27 patients showed multiple patchy ground-glass shadows in the lungs. Three patients had elevated liver enzymes and nine developed fetal distress. In 17 patients who had detailed treatment data, all patients received oxygen support via a nasal cannula, 14 patients underwent antiviral therapy, and 16 patients underwent antibiotic treatment. As a result, 26 patients delivered via cesarean section and two patients via vaginal delivery. All neonates survived except for one. It should be noted that in the study by Zhu et al. some neonates born to mothers with COVID-19 had adverse neonatal outcomes such as premature labor, respiratory distress, thrombocytopenia accompanied by abnormal liver function, and even death (Zhu et al., 2020).

With regard to SARS, 19 cases have been reported during pregnancy (Zhang et al., 2003; Robertson et al., 2004; Wong et al., 2004; Yudin et al., 2005). Seven patients became infected in the first trimester, five patients in the second trimester, and seven patients in the third trimester. The most common symptoms of infected pregnant women included fever (100%), cough (78.9%), and myalgia (68.4%). Lymphocytopenia was observed in 15 pregnant women, and chest radiographs of all pregnant women indicated viral infection in the lungs. Twelve pregnant women were given antiviral drugs, 14 pregnant women were given antibiotic drugs, and 12 pregnant women were given corticosteroids. Spontaneous miscarriage was observed in four out of seven patients who were infected in the first trimester. Three of the 19 pregnant women died; however, all the neonates who were delivered in the second or third trimester survived. Two cases suffered from fetal growth restriction and oligohydramnios, four cases developed fetal distress, and there was one case of spontaneous preterm birth.

Eleven cases of pregnancy with MERS were reported (Payne et al., 2014; Alserehi et al., 2016; Assiri et al., 2016; Malik et al., 2016; Park et al., 2016; Alfaraj et al., 2019). One patient was infected in the first trimester, five patients in the second trimester, and six patients in the third trimester. Most pregnant women presented with dyspnea (72.7%), cough (63.6%), and fever (54.5%); their chest radiographs revealed typical signs of viral infection. Unfortunately, there were no detailed treatment-related data. The woman infected in the first trimester continued pregnancy after her virus nucleic acid testing for MERS-Cov turned negative. Two patients had stillbirths, and one patient developed placental abruption. Three pregnant women and three neonates died.

## Diagnosis and Treatment of COVID-19 in Pregnancy

The diagnosis of COVID-19 in pregnancy is mainly based on epidemiological history, clinical manifestations, chest radiography, and etiological tests (National Health Commission of the People's Republic of China, 2020). This is similar to the evidence of diagnosis for SARS and MERS in pregnancy. According to our literature review, most pregnant women infected by the three coronaviruses had an epidemiological contact history. Fever, cough, myalgia, and dyspnea are the major symptoms. CT images revealed typical signs of viral infection in the lungs. Nasopharynx swab or sputum was positive in the virus nucleic acid test. Lymphopenia and elevated liver enzymes were commonly seen in pregnant women with COVID-19 and SARS.

According to the seventh guidelines published by the National Health Commission of China, the treatment of COVID-19 mainly involves oxygen therapy, antiviral therapy, and supportive treatment (National Health Commission of the People's Republic of China, 2020). At present, there is no antiviral drug for the treatment of COVID-19; therefore, identifying an antiviral drug against COVID-19 in pregnancy is imperative.

Interferon-alpha is a broad-spectrum antiviral drug that activates antiviral protein genes and modulates immune cell function (Totura and Bavari, 2019). For SARS-CoV-1 and MERS-CoV, interferon-alpha was effective at reducing viral replication *in vitro* and *in vivo* (Cinatl et al., 2003; Falzarano et al., 2013a,b). Regarding its safe use in pregnant women, one systematic review suggested that interferon-alpha does not significantly increase the risk of adverse perinatal outcomes above general population rates (Yazdani Brojeni et al., 2012).

Lopinavir/ritonavir is a human immunodeficiency virus (HIV) protease inhibitor that was found to target the SARS-CoV-1 nonstructural protein 3CLpro *in vitro* (Lu, 2020). During the SARS-CoV-1 epidemic, lopinavir/ritonavir combined with ribavirin reduced the viral load and decreased the rate of death or ARDS (Chu et al., 2004). Lopinavir/ritonavir also increased the viral clearance rate and survival rate in patients with MERS (Chan et al., 2015). Lopinavir/ritonavir was also administered to pregnant women with HIV. It was found that HIV proliferation was effectively suppressed during pregnancy and through 1 year postpartum (Cohan et al., 2015). Furthermore, the rate of preterm deliveries, low-birth-weight neonates, stillbirths, and birth defects did not increase (Roberts et al., 2009; Cohan et al., 2015).

In addition, ribavirin and favipiravir are representative of a guanosine analog. Mechanically, ribavirin and favipiravir can inhibit virus RNA synthesis by viral RNA-dependent RNA polymerase and inhibit mRNA capping (Totura and Bavari, 2019). In the previous literature, ribavirin was administered to pregnant women with SARS. However, it was found that ribavirin at the concentration of typical human regimens could not significantly inhibit coronavirus replication *in vitro* (Shen et al., 2016). Furthermore, meta-analyses have found that its role was limited in the treatment of coronavirus-related respiratory syndromes (Stockman et al., 2006; Morra et al., 2018). The resistance might be explained by nsp14 exoribonuclease being expressed by coronavirus. As nsp14 exoribonuclease can function as an excision nucleoside analog due to its proofreading activity (Minskaia et al., 2006), it is speculated that COVID-19 might also be resistant to ribavirin or favipiravir (Chan et al., 2020).

In contrast, remdesivir, a nucleoside analog, could act against both SARS-CoV-1 and MERS-CoV *in vitro* (Sheahan et al., 2017). *In vivo*, remdesivir was more effective than lopinavir/ritonavir combined with interferon-β in decreasing the viral load of mice infected with MERS-CoV (Sheahan et al., 2020). The mechanism involved is that it can interfere with the nsp12 polymerase even in the setting of nsp14 exoribonuclease proofreading activity (Agostini et al., 2018). As expected, remdesivir was proved to be effective in the inhibition of SARS-CoV-2 replication *in vitro* (Wang et al., 2020a). However, data about its safety during pregnancy are absent.

Initially, chloroquine was used as an antimalarial drug. It was later found to act against SARS-CoV-1 in primate cells either before or after exposure to the virus (Vincent et al., 2005). Recently, chloroquine was found to inhibit the proliferation of SARS-CoV-2 *in vitro* (Wang et al., 2020a). The mechanism involved might be that it interferes with terminal glycosylation of ACE2 (Vincent et al., 2005). As to its safety in pregnancy, chloroquine was not shown to be associated with birth weight, gestational age, growth, or development of newborns, neurological development, and visual acuity in infants in the previous literature (Villegas et al., 2007; Ward et al., 2007; Divala et al., 2018).

Arbidol, which is a broad-spectrum antiviral drug whose mechanism involves inhibition of enveloped virus membrane fusion, has also been used (Blaising et al., 2014). Arbidol and arbidol mesylate were effective in suppressing the proliferation of SARS-CoV-1 *in vitro* (Khamitov et al., 2008). Furthermore, arbidol was effective against SARS-CoV-2 *in vitro* (Wang et al., 2020b). In Russia, arbidol was administered to pregnant women with influenza (Bulgakova et al., 2017). In our the studies included in our literature review, arbidol was found to have been administered to pregnant women with COVID-19 (Yu et al., 2020; Zhao et al., 2020). However, its safety in pregnancy requires further research.

In summary, interferon-alpha, lopinavir/ritonavir, and chloroquine could be administered to pregnant women with COVID-19 after patients were fully informed of these drugs' benefits and risks. Remdesivir and arbidol are promising antiviral drugs against COVID-19; however, their safety in pregnancy requires further research. In addition, antibiotics and corticosteroids are recommended in pregnancy with COVID-19 if necessary (Mer and Richards, 1998; Wong et al., 2003). However, antibiotics should not be used without indication, and high doses of glucocorticoids should be avoided as much as possible (National Health Commission of the People's Republic of China, 2020).

## Perinatal Outcomes of COVID-19 in Pregnancy

Due to impaired cellular immunity and physiological changes, mild pneumonia is more likely to develop into severe pneumonia in pregnant women, and severe pneumonia is deleterious for the fetus (Lam et al., 2004; Goodnight and Soper, 2005). Thus, timely pregnancy termination may be beneficial for both mothers and infants if the disease advances. As to the mode of pregnancy termination, cesarean section is recommended (National Health Commission of the People's Republic of China, 2020). However, if the pregnant woman's condition does not worsen, there is a chance of having a healthy baby if pregnancy is continued. In the previous literature, pregnant women with SARS or MERS who continued pregnancy after their etiological test turned negative had favorable maternal and neonatal outcomes. However, some of them had suffered intrauterine growth restriction (IUGR), meconium-stained amniotic fluid, or fetal distress.

Pathological examination of placentas from women with COVID-19 showed various degrees of fibrin deposition inside and around the villi with local syncytial nodules, concomitant with the morphology of chorionic hemangioma or massive placental infarction (Chen S. et al., 2020). Further, the results from SARS-related pregnancies revealed increased intervillous, subchorionic fibrin, or extensive fetal thrombotic vasculopathy (Ng et al., 2006). It was speculated that these placental pathological morphological changes were caused by hypoxia during coronavirus infection, and they may result in IUGR, meconium-stained amniotic fluid, or fetal distress. Similarly, miscarriage and stillbirth could also result from hypoxia caused by coronavirus infection. Therefore, pregnant women with COVID-19 should be closely monitored, even after their nucleic acid test for SARS-Cov-2 turns negative.

In the studies included in our literature review, all pregnant women with COVID-19 and their neonates survived except for one neonate (Zhu et al., 2020). However, several pregnant women with SARS or MERS died or had a stillbirth, and some neonates failed to survive. It is speculated that COVID-19 during pregnancy might be less severe than SARS or MERS during pregnancy. This is consistent with the conclusion that the mortality rate of COVID-19 is less than that of SARS or MERS (Han et al., 2020; Liao et al., 2020).

Amniotic fluid, cord blood, placenta, and the fetal membrane from pregnant women with COVID-19, SARS, and MERS were all negative when tested with a viral nucleic acid test for SARS-CoV-2, SARS-CoV-1, and MERS-CoV, respectively, in the studies included in our literature review. This could be partly explained by the different distribution of their cellular receptors. The ACE2 protein is mainly expressed on lung alveolar epithelial cells and enterocytes of the small intestine (Hamming et al., 2004). However, ACE2 mRNA expression was found to be low in cells from the early maternal-fetal interface with single-cell RNA-sequencing technology. Hence, it is speculated that the ratio of SARS-CoV-2-infected mother-to-fetus transmission will be relatively low (Zheng et al., 2020). Even if SARS-CoV-2 could not transmit from mothers to babies, it could be transmitted by close contact because coronavirus also exists in the sweat glands and the distal convoluted tubules of the kidney in addition to the intestinal and respiratory tracts (To and Lo, 2004; Zhou et al., 2017). Therefore, the neonates of pregnant women with COVID-19 should be isolated for at least 14 days and should not be breastfed (National Health Commission of the People's Republic of China, 2020).

## Conclusion

This research has provided some strategies for the obstetric management of pregnant women with COVID-19. To some extent, clinical characteristics were similar among pregnancies with COVID-19, SARS, and MERS. It seems that COVID-19 during pregnancy is milder than SARS and MERS during pregnancy. However, pregnant women with COVID-19 should be closely monitored, even after their etiological tests turn negative. Maternal separation is necessary even if there was no vertical transmission. Interferon-alpha, lopinavir-ritonavir, and chloroquine could be good candidates for the treatment of COVID-19 during pregnancy, but more work is needed to explore their effectiveness and safety. In addition, remdesivir and arbidol, which are potential drug treatments for COVID-19, require further research on their safety during pregnancy.

## Author Contributions

YL and DL provided the concept. YM drafted the manuscript. SW, XL, and YP revised the manuscript. XY collected the data. All authors approved the final version for publication.

## Conflict of Interest

The authors declare that the research was conducted in the absence of any commercial or financial relationships that could be construed as a potential conflict of interest.

## Abbreviations

GA: gestational age
COVID-19: 2019 novel coronavirus disease
SARS: severe acute respiratory syndrome coronavirus
MERS: Middle East Respiratory Syndrome coronavirus
CoV: coronavirus
ACE2: angiotensin-converting enzyme 2
HIV: human immunodeficiency virus
CT: computed tomography
CTG: cardiotocography
PROM: premature rupture of membranes
DIC: disseminated intravascular coagulation
IUGR: intrauterine growth retardation
HIV: human immunodeficiency virus
N/A: not available.
